# Development of a core dataset for child injury surveillance: a modified Delphi study in China

**DOI:** 10.3389/fped.2023.970867

**Published:** 2023-04-28

**Authors:** Hairong Gong, Yuan Wang, Yongzhen Li, Pengpeng Ye, Li Xie, Guoping Lu, Jing Liu, Jun Song, Xiaowen Zhai, Hong Xu, Leilei Duan

**Affiliations:** ^1^ Emergency Department Children's Hospital of Fudan University, Shanghai, China; ^2^ National Center for Chronic and Non-Communicable Diseases Control and Prevention, Chinese Center for Disease Control and Prevention, Beijing, China; ^3^ Department of Clinical Nutrition New Hong Qiao Campus for Children's Hospital of Fudan University, Shanghai China; ^4^ Clinical Research Institute School of Medicine, Shanghai Jiao Tong University, Shanghai, China

**Keywords:** child injury, injury surveillance, modified Delphi method, core dataset, surveillance tool

## Abstract

**Background:**

Understanding the occurrence and severity of child injuries is the cornerstone of preventing child injuries. Currently, there is no standardized child injury surveillance dataset in China.

**Methods:**

Multistage consultation by a panel of Chinese experts in child injury to determine items to include in the core dataset (CDS) was performed. The experts participated in two rounds of the modified Delphi method comprising a consultation questionnaire investigation (Round 1) and a face-to-face panel discussion (Round 2). Final consensus was established based on the opinions of the experts regarding the modified CDS information collection items. Enthusiasm and authority exhibited by the experts were evaluated by the response rate and using the expert authority coefficient, respectively.

**Results:**

The expert panel included 16 experts in Round 1 and 15 experts in Round 2. The experts during both rounds had a high degree of authority, with an average authority coefficient of 0.86. The enthusiasm of the experts was 94.12%, and the proportion of suggestions reached 81.25% in Round 1 of the modified Delphi method. The draft CDS evaluated in Round 1 included 24 items, and expert panelists could submit recommendations to add items. Based on findings in Round 1, four additional items, including nationality, residence, type of family residence, and primary caregiver were added to the draft of the CDS for Round 2. After Round 2, consensus was reached on 32 items arranged into four domains—general demographic information, injury characteristics, clinical diagnosis and treatment, and injury outcome—to include in the final CDS.

**Conclusion:**

The development of a child injury surveillance CDS could contribute to standardized data collection, collation, and analysis. The CDS developed here could be used to identify actionable characteristics of child injury to assist health policymakers in designing evidence-based injury prevention interventions.

## 1. Introduction

Child injury is the main cause of childhood hospitalization and death worldwide and has become an increasingly severe public health problem globally (1). More than 98% of child injury deaths occur in developing countries (2). In China, mortality for children under 18 years old was 11.42/100,000 in 2019 (3), driven by injury-related deaths, which are the leading cause of death for children under 6 years of age (1).

To effectively and systematically prevent child injuries, the first fundamental step is to understand factors surrounding their occurrence as well as factors that influence their severity (4, 5). The Injury Surveillance Systems help determine injury patterns and severity through continuous and systematic data collection, analysis, interpretation, and dissemination, as well as by providing necessary evidence to prescribe preventive measures. Although the National Injury Surveillance System (NISS) had been established in China since 2003, the all-age-grouped dataset lacks clinical characteristics and child growth and development information. Furthermore, most previous studies are either from non-tertiary hospitals focusing on the basic epidemiological characteristics of injury (6–8) or from general hospitals focusing on the disease burden collected in the all-age-grouped database (9–11). It is known that distinct behaviors and growth patterns in children cause the characteristics of injuries in children to be quite different from those in adults (12), creating an urgent need for the development of a standardized core dataset (CDS) for child injury surveillance. Establishing a more practical and routinely applied surveillance CDS for emergency departments is therefore essential for the collection of high-quality data on child injuries (13).

This study developed a surveillance CDS that is better suited to the characteristics and patterns of child injury and is more cohesive with emergency medical practice to provide insight into child injury prevention toward developing targeted strategies to control child injury.

## 2. Methods

**Figure 1:**
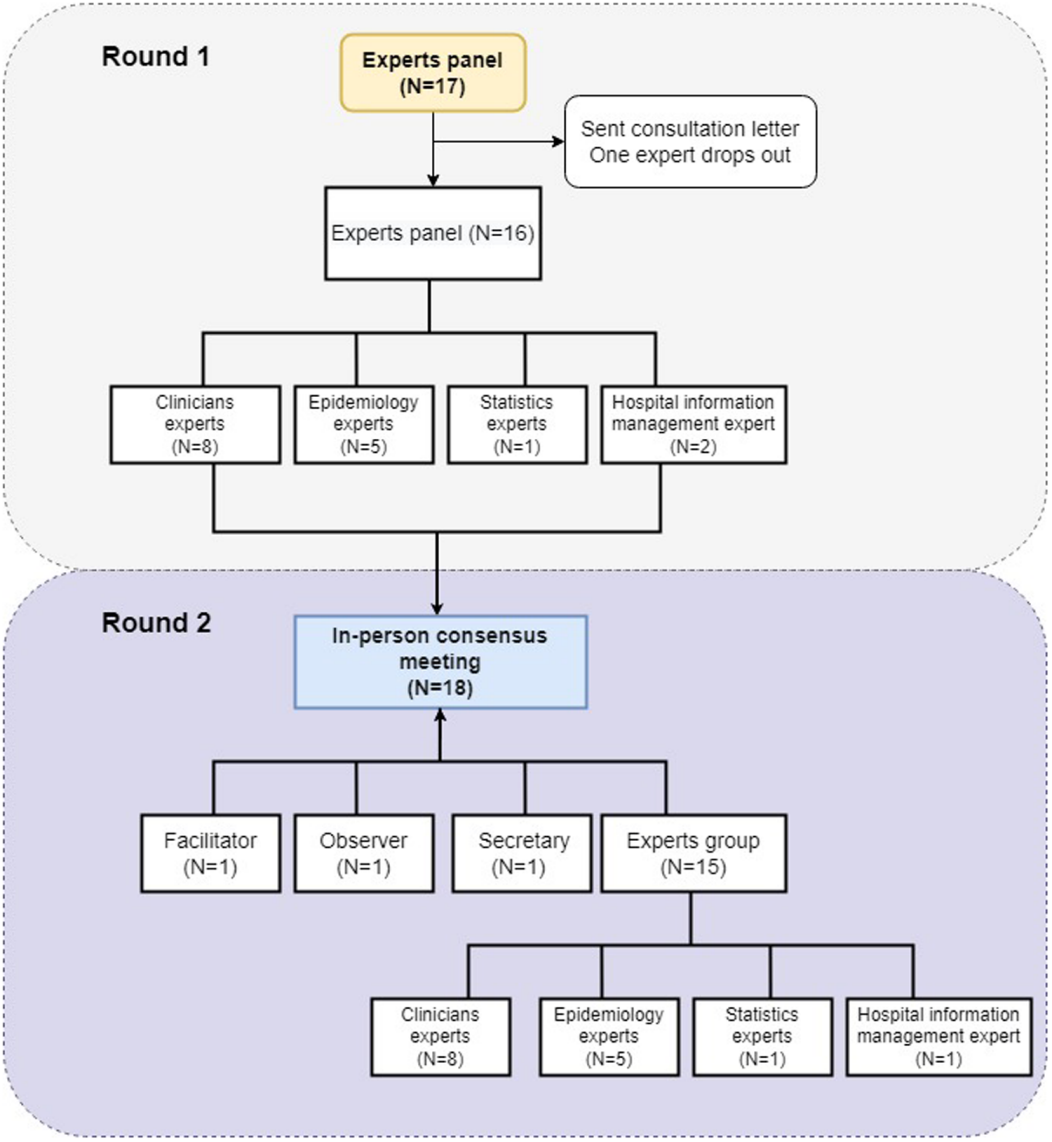
Flow chart of the modified Delphi method used to develop the Child Injury Surveillance CDS. Gray and purple indicate Round 1 and Round 2 of the Modified Delphi study, respectively.

A modified Delphi study is a structured, questionnaire-based method for converting the opinions of multiple experts into a consensus. Recently, the modified Delphi method has gained popularity in medical research. In this study, a core study group designed a two-round modified Delphi study, which was conducted between June 2020 and January 2021, to achieve consensus on a CDS for child injury surveillance (Figure 1). The core study group comprised five members involved in this project who were not among the experts who served as panelists for the modified Delphi study.

### 2.1. Expert panel selection

A multidisciplinary expert panel to develop the CDS was recruited using an academic reputation-based snowball sampling method. The inclusion criteria for members of the expert panel were (1) clinicians whose work had been related to the child injury disease field for >5 years and who were familiar with the clinical characteristics and prognoses of child injuries; (2) researchers who engaged in epidemiological research of child injury or in research related to prevention and control of injuries for the Center for Disease Control and Prevention Bureau; (3) biostatisticians with ≥5 years of experience in public health; or (4) information technology engineers with >5 years of experience managing Hospital Information Systems.

### 2.2. Modified Delphi study design

Creation of the injury surveillance CDS involved three main steps: (1) collect and determine candidate items that could be included in the CDS of child injury surveillance; (2) validate the chosen items by collecting expert opinions using a questionnaire (Round 1); and (3) convene a meeting of experts to discuss the revised surveillance CDS and determine the final CDS items (Round 2). During conduction of this study, we followed the basic principles of the Delphi method: anonymous rating, multiple rounds of consultation, and providing feedback to participants before starting the next round of consultation.

#### 2.2.1. Step 1: identify candidate items for possible inclusion in the child injury surveillance CDS

The core study group used information from a literature review (14–18) and from combing of the current NISS dataset in China early during the project (19) and combined this with the epidemiological and clinical characteristics of child injury to create a first draft of items to include in the CDS for the Delphi expert consultation questionnaire. In accordance with the World Health Organization (WHO) and Center for Disease Control Injury Surveillance Guidelines, fundamental characteristics of an injury surveillance system, such as simplicity, flexibility, acceptability, reliability, utility, sustainability, and timeliness, were thoroughly considered when creating the draft (20).

#### 2.2.2. Step 2: Round 1—expert panel consultation questionnaire

A consultation questionnaire was sent to the panel of experts between October 2020 and November 2020 via email. Expert consultants were given project guidance and asked to complete the consultation questionnaire. The project guidance presented the context, objective, scope, and significance of the project and consultation as well as provided a concise explanation of the significance and value of expert opinions. The consultation questionnaire contained the fundamental information of experts, drafted questions for capturing child injury surveillance items in the CDS, and self-evaluations of experts' familiarity with consulting problems and evaluation criteria. Experts' familiarity was rated on a five-level scale, and the evaluation was based on seven factors, including epidemiology, clinic, statistics, and monitoring systems with varying quantitative values. The importance of each item in the consultation questionnaire was rated on a 5-point Likert scale, where 5 = very important, 4 = important, 3 = moderately important, 2 = unimportant, and 1 = very unimportant. Meanwhile, opinion boxes were set under each item for experts to write in suggestions regarding the items.

#### 2.2.3. Step 3: Round 2—expert panel consultation meeting

Before Round 2, the core group modified the items in the draft CDS according to the opinions collected from the expert group in Round 1, and the new draft CDS was provided to the expert panel for discussion during an in-person meeting held in January 2021. The final CDS items reflect the votes of the expert panel.

### 2.3. Statistical methods

Demographic characteristics of the expert panelists were collected and analyzed. The frequencies, percentages, interquartile ranges, and medians of expert responses from the Round 1 questionnaire were calculated to determine the consistency of the experts on each item. If the collective consistency of adding a new item had a mean importance index >3.75 points (75% of the 5-point Likert scale) or deleting an item had a mean importance index <3.75 points, the opinion was accepted and the corresponding item was modified before Round 2. The expert positivity index was calculated as the response rate on the expert consultation questionnaire, and >70% response rate was interpreted as high motivation. The authoritative indicators of the experts were expressed by the coefficient of reliability (Cr), which was represented by the results of the self-evaluations of the experts. Cr was determined using the coefficient of adjudication (Ca) and coefficient of sense (Cs), which reflect the familiarity of the experts with the consulting content, using the equation Cr = (Ca + Cs)/2. Cr values ≥0.7 were considered acceptable (21, 22). The coefficient of adjudication (Ca) was composed of four coefficients: research evidence, practical experience, clinical guidelines, and instinct. Different weighting scales were assigned to each item according to its influence intensity. All statistical analyses were performed using Stata 13.0 software (Stata Corporation, College Station, TX, United States). GraphPad Prism 8.0.1 (GraphPad Software, San Diego, CA, United States) was used to plot the data.

## 3. Results

### 3.1. Demographic characteristics of the expert panel

We invited 18 experts to participate in two rounds of the Delphi method as part of an expert panel. In Round 1, 16 experts participated, and 15 experts participated in Round 2. Clinicians, injury epidemiologists, health statisticians, and information technology engineers were all represented on the expert panel during both rounds. Members in the expert panel were from a wide range of provinces in China, including Beijing, Shanghai, Guangdong, Zhejiang, and Shaanxi, with relatively equal numbers of men and women (Supplementary Table S1). All of the experts had at least a master's degree, and 56.3% (9/16) and 66.7% (10/15) of the experts in Round 1 and Round 2, respectively, had a doctoral degree. The number of years of work experience of the experts ranged from 5 to 30 years, with 18.3 and 21.1 average working years of experience among the experts in Round 1 and 2, respectively. Table 1 and Supplementary Table S1 present the demographic characteristics of the expert panelists during both rounds of the modified Delphi study.

**Table 1:** Demographic characteristics of modified Delphi expert panelists who participated in developing the child injury surveillance CDS.

Demographic characteristics	Round 1	Round 2
	(*N *= 16)	(*N *= 15)
**Sex, *n* (%)**		
Male	8 (50.0)	6 (40.0)
Female	8 (50.0)	9 (60.0)
**Education level, *n* (%)**		
Master's degree	7 (43.8)	5 (33.3)
Doctoral degree	9 (56.3)	10 (66.7)
Time as an expert (years), mean (SD)	18.3 (9.4)	21.1 (8.0)
**Field of expertise, *n* (%)**		
Clinical	8 (50.0)	8 (53.3)
Injury epidemiology	5 (31.3)	5 (33.3)
Health statistics	1 (6.3)	1 (6.7)
Hospital information management	2 (12.5)	1 (6.7)

CDS, core dataset; SD, standard deviation.

### 3.2. Implementation and reliability analysis

An implementation and reliability analysis was conducted by evaluating the authority of experts (Supplementary Tables S2, S3). Overall, 17 questionnaires were sent out and 16 were collected at the end of Round 1, yielding an overall response rate of 94.11%. Among the experts who returned the questionnaires, 13 (81.25%) experts provided additional suggestions. The authority coefficient of each expert was calculated by assigning different-weight coefficients to academic degree held by the expert. The Cr of the experts in this study ranged from 0.625–1.0, and the mean Cr of the 16 experts who participated in Round 1 was 0.86 (SD = 0.10) (Table 2), indicating a high degree of authority among the expert panel.

**Table 2:** Quantitative assessment of familiarity of modified Delphi study panelists with child injury.

Expert panelist identifier number	Research evidence	Practical experience	Clinical guidelines	Instinct	Coefficient of adjudication (Ca)	Coefficient of sense (Cs)	Coefficient of reliability (Cr)
01	0.40	0.30	0.15	0.05	0.90	0.55	0.73
02	0.50	0.30	0.15	0.05	1.00	0.75	0.88
03	0.50	0.30	0.15	0.05	1.00	0.70	0.85
04	0.50	0.30	0.15	0.05	1.00	0.70	0.85
05	0.50	0.30	0.15	0.05	1.00	1.00	1.00
06	0.40	0.30	0.15	0.05	0.90	0.95	0.93
07	0.50	0.30	0.15	0.05	1.00	0.80	0.90
08	0.40	0.30	0.15	0.05	0.90	0.80	0.85
09	0.40	0.30	0.15	0.05	0.90	0.80	0.85
10	0.50	0.30	0.15	0.05	1.00	0.90	0.95
11	0.40	0.20	0.15	0.05	0.80	0.90	0.85
12	0.40	0.20	0.15	0.05	0.80	0.60	0.7
13	0.50	0.20	0.15	0.05	0.90	0.35	0.63
14	0.50	0.30	0.15	0.05	1.00	0.80	0.90
15	0.50	0.30	0.15	0.05	1.00	0.95	0.98
16	0.50	0.30	0.15	0.05	1.00	0.90	0.95
Mean (SD)	0.46 (0.05)	0.28 (0.04)	0.15 (0)	0.05 (0)	0.94 (0.07)	0.78 (0.17)	0.86 (0.10)

SD, standard deviation.

### 3.3. Determination of items to include in the draft CDS

A draft CDS of Child Injury Surveillance was created by integrating data mined by the core study group to create a four-part document that included a total of 24 items. Part I collected eight items regarding general demographic information of the children and their parents or guardians, including (1) outpatient admission number, (2) sex, (3) date of birth, (4) weight, (5) height, (6) residence, (7) nationality, and (8) parent education level. Part II collected eight items regarding the characteristics of the injury, including (1) time of injury, (2) time of hospital visit, (3) mechanism of injury, (4) location of injury, (5) activity at the time of the injury, (6) intention of the injury, (7) caregiver type, and (8) caregiver status. Part III collected four items regarding clinical diagnosis and treatment of the injury, including (1) nature of the injury, (2) body parts affected by injuries, (3) body systems affected by injury, and (4) the Pediatric Trauma Score (PTS), which can easily, quickly, and accurately identify the severity of traumatic injuries in children. Part IV collected four items regarding the outcome of the injury, including (1) severity of the injury, (2) clinical diagnosis, (3) injury outcome, and (4) date of injury outcome. Meanwhile, four additional administrative items were collected, including (1) the monitoring hospital number, (2) report card number, (3) reporter, and (4) date.

### 3.4. Two rounds of the modified Delphi method

#### 3.4.1. Modified Delphi Round 1

**Figure 2:**
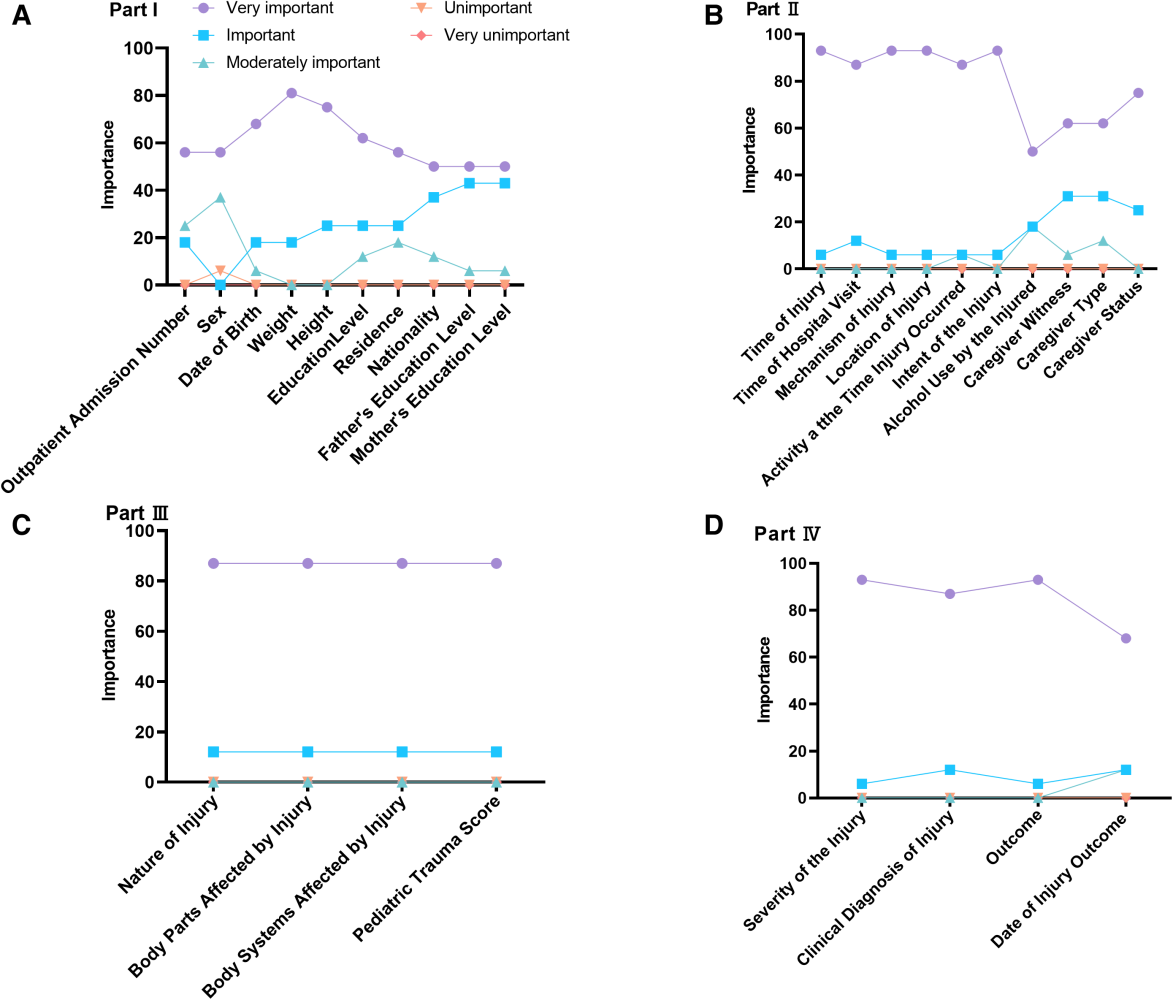
Importance of evaluating items in the draft child injury surveillance CDS based on expert opinion. (**A**) Part I: General patient information; (**B**) Part II: Basic injury information; (**C**) Part III: Clinical injury information; (**D**) Part IV: Injury outcome information.

A flow chart depicting the modified Delphi method used in this study is provided in Figure 1. The expert panel comprehensively evaluated the importance and rationality of each of the 28 items in the draft CDS and provided detailed suggestions regarding the deletion and improvement of items. For Part I, the most highly rated demographic items were sex (81%), date of birth (75%), outpatient admission number (69%), weight (63%), and medical number (63%). The experts suggested that the CDS collects information of child caregiver educational background, age, intellectual development, education level or school status of the child, siblings, current residence, race, etc. In Part II, the panel determined that the most important characteristics of the injury to collect were injury time, reason, location, time of patient's visit, and the activity when the injury occurred, all of which scored >88%. The status of the caregiver at the time of injury also scored highly (75%), followed by the primary family caregiver (63%) and the main caregiver at the time of injury (63%). Whether alcohol use was involved in the injury scored the lowest among the items in Part II (50%). All four of the items in Part III were considered very important or important by the experts, reflecting the high relevance of collecting adequate data regarding the clinical diagnosis and treatment of the injury. In Part IV, the panel determined that the most important items related to injury outcome were severity of injury (94%), outcome of injury (94%), clinical diagnosis of injury (88%), and occurrence date of outcome of injury (69%) (Figure 2). The draft CDS of Child Injury Surveillance was revised based on this feedback from Round 1, which included adding ethnicity, place of residence, family residence type, and child's primary caregiver to patient demographic information.

#### 3.4.2. Modified Delphi Round 2

Following an interview schedule prepared in advance, we conducted Round 2 of the modified Delphi study as an online, in-person consensus meeting for 3 h in January 2021. An observer and a secretary were present to document the interactions among the expert panel and facilitator. There was no conflict of interest for any of the expert panelists nor for the facilitator, observer, or secretary. At this meeting, 30 proposed items for the CDS were evaluated. After extensive discussion, 32 final core items were included in the CDS of Child Injury Surveillance (Table 3).

**Table 3:** Items included in final version of Child Injury Surveillance CDS.

No.	Domain of information	Core dataset items
1	*Part I: general demographic information*	Outpatient Admission Number
2	* *	Sex
3	* *	Date of Birth
4	* *	Weight
5	* *	Height
6	* *	Education Level
7	* *	Residence
8	* *	Nationality
9	* *	Father's Education Level
10	* *	Mother's Education Level
11	*Part II: basic injury information*	Time of Injury
12	* *	Time of Hospital Visit
13	* *	Mechanism of Injury
14	* *	Location of Injury
15	* *	Activity at the Time Injury Occurred
16	* *	Intent of the Injury
17	* *	Alcohol Use by the Injured
18	* *	Caregiver Witness
19	* *	Caregiver Type
20	* *	Caregiver Status
21	*Part III: clinical injury information*	Nature of Injury
22	* *	Body Parts Affected by Injury
23	* *	Body Systems Affected by Injury
24	* *	Pediatric Trauma Score (PTS)
25	*Part IV: injury outcome information*	Severity of the Injury
26	* *	Clinical Diagnosis
27	* *	Injury Outcome
28	* *	Date of Injury Outcome
29	*Administrative information*	Monitoring Hospital Number
30	* *	Report Card Number
31	* *	Reporter
32	* *	Date

CDS, core dataset.

## 4. Discussion

Because injuries to children differ from those in adults, precise information related to child-specific injuries, such as activity at the time of injury, guardian status, and injury characteristics, are needed to develop measures to prevent child injuries. Previous studies have shown that children of different ages are vulnerable to different injury types or injury places, and that the characteristics of injuries also differ according to age (7, 23, 24). For example, children of primary school age are vulnerable to bite injuries, whereas the proportions of traffic injuries or suicide increase between 15 and 17 years of age, and accidental injuries such as falls are more common in infants (25). Therefore, tailored prevention and policy intervention are needed to reduce a wide array of child injuries. Here, we developed a CDS of Child Injury Surveillance by amending a draft CDS on the basis of expert opinions and suggestions that can offer comprehensive and uniform data collection to create a robust dataset that may be used to understand and prevent child injury.

Previous studies have reported that occupation and income level of families are correlated with child injury (26, 27); thus, collecting residence location could reflect socioeconomic status and inform on whether these factors may indeed influence child injury. Studies have also shown that unintentional child injuries may be reduced by parents teaching safety rules (28, 29). Also, the parents' level of education can influence the security of a child's environment, including safety standards and level of supervision, which should be significant in determining child injuries. Therefore, we included the education level of the parents/guardians as relevant demographic information in the final CDS to allow investigating the correlation between the parent educational level and injury characteristics and to promote targeted prevention.

Traditional and modified Delphi methods are often used in medical and health services research (30–32) to facilitate effective decision making in situations where conflicting or inadequate information exists. This study is the first to use the modified Delphi method to develop a child-specific injury surveillance CDS. By improving and refining the system for multidepartment, coordinated data collection regarding child injuries, the CDS provides a systematic, comprehensive, scientific, and accurate mechanism to improve our understanding of the characteristics of child injury to design prevention strategies.

Our study has some limitations. First, the CDS would require additional work from emergency department staff who already have a heavy work load. With this in mind, we meticulously designed and reviewed the CDS items, and the majority of them can be extracted automatically from the electronic health record system. Second, we did not include the specific information that resides within each of the included items, such as expansion of the mechanism (reason, cause) code to the second level of detail, which would enhance the understanding of the problem and thus inform prevention strategies. We intend to introduce additional datasets containing comprehensive information on child injury surveillance in the future. Third, an important study limitation is that the panel only included experts from China, while an international panel would have been preferable. Hence, this may impact the generalizability of the CDS.

Injury is an important public health problem that endangers child health. The surveillance of child injury and various epidemiological studies have contributed to preventing child injury and reducing disease burden. However, retrospective information collected by questionnaires often has recall bias, and memory discrepancies may also result in errors in the recall of guardians or teachers. As treatment of child injury is mainly in the outpatient or emergency settings, establishing this standardized, integrated child injury surveillance dataset and using it in hospital emergency departments can capture demographic and clinical characteristics of child injury. Consequently, the CDS may contribute to tailored intervention strategies to prevent child injury by systematically collating comprehensive, unbiased data to identify at-risk populations.

## 5. Conclusion

In conclusion, we developed a child injury surveillance CDS using the modified Delphi method and achieved a multidisciplinary, multiexpert consensus. The child injury surveillance CDS could provide a foundation for establishing standardized and high-quality child injury data collection, collation, and analysis to identify actionable characteristics of child injury that can assist health policymakers in designing evidence-based injury prevention interventions.

## Data Availability

The raw data supporting the conclusions of this article will be made available by the authors, without undue reservation.
