# Supplementary material for: Development of a core dataset for child injury surveillance: a modified Delphi study in China
Source: Front Pediatr. 2023 Apr 28;11:970867. doi: 10.3389/fped.2023.970867 (PMC10175816; doi:10.3389/fped.2023.970867)
Supplement: Supplementary file 1 [file Table1.docx]

| **Supplementary Table S1 Basic characteristics of panel lists for development of child injury Core dataset in the Modified Delphi Methods in round 1 and round 2** | | | | | | |  | |  |
| --- | --- | --- | --- | --- | --- | --- | --- | --- | --- |
| **Number** | **Location** | **Gender** | **Degree of education** | **Title** | **Professional** **fields** | **Expert experience(years)** | | **Round** | |
| 1 | Guangdong | Male | Master | Chief physician | Pediatric surgery | 30 | | 1&2 | |
| 2 | Zhejiang | Male | Master | Researcher | Injury epidemiology | 12 | | 1&2 | |
| 3 | Beijing | Male | Doctor | Deputy chief physician | Injury epidemiology | 20 | | 1&2 | |
| 4 | Shanghai | Female | Master | Researcher | Injury epidemiology | 10 | | 1&2 | |
| 5 | Shanghai | Female | Doctor | Professor | Injury epidemiology | 30 | | 1&2 | |
| 6 | Guangdong | Female | Master | Researcher | Injury epidemiology | 12 | | 1&2 | |
| 7 | Shanghai | Female | Master | Deputy chief physician | Hospital information technology | 15 | | 1&2 | |
| 8 | Shanghai | Male | Doctor | Associate professor | Health statistics in public health | 11 | | 1&2 | |
| 9 | Beijing | Male | Doctor | Chief physician | Emergency medicine | 30 | | 1&2 | |
| 10 | Zhejiang | Female | Doctor | Chief physician | Emergency medicine | 30 | | 1&2 | |
| 11 | Henan | Female | Doctor | Chief physician | Emergency medicine | 28 | | 1&2 | |
| 12 | Beijing | Female | Doctor | Chief physician | Emergency medicine | 18 | | 1&2 | |
| 13 | Shaanxi | Male | Doctor | Chief physician | Child orthopedics | 26 | | 1&2 | |
| 14 | Shanghai | Male | Master | Associate Researcher | Injury epidemiology | 10 | | 1 | |
| 15 | Shanghai | Male | Master | Lecturer | Hospital information technology | 5 | | 1 | |
| 16 | Shanghai | Female | Doctor | Deputy chief physician | Emergency medicine | 5 | | 1 | |
| 17 | Chongqing | Female | Doctor | Chief physician | Emergency medicine | 16 | | 2 | |
| 18 | Shanghai | Female | Doctor | Deputy chief physician | Emergency medicine | 28 | | 2 | |
