# Supplementary material for: Development of a core dataset for child injury surveillance: a modified Delphi study in China
Source: Front Pediatr. 2023 Apr 28;11:970867. doi: 10.3389/fped.2023.970867 (PMC10175816; doi:10.3389/fped.2023.970867)
Supplement: Supplementary file 2 [file Table2.docx]

**Supplementary Table S2 Weighting scale of items in the Coefficient of adjudication (Ca) in the modified Delphi method**

| **Adjudication** |  | **Influence** |  |
| --- | --- | --- | --- |
|  | **Strong** | **Medium** | **Weak** |
| Research evidence | 0.50 | 0.40 | 0.30 |
| Practical experience | 0.30 | 0.20 | 0.10 |
| Clinical guidelines | 0.15 | 0.15 | 0.15 |
| Intuitive instinct | 0.05 | 0.05 | 0.05 |
| Total | 1.00 | 0.80 | 0.60 |
