# Supplementary material for: Development of a core dataset for child injury surveillance: a modified Delphi study in China
Source: Front Pediatr. 2023 Apr 28;11:970867. doi: 10.3389/fped.2023.970867 (PMC10175816; doi:10.3389/fped.2023.970867)
Supplement: Supplementary file 3 [file Table3.docx]

**Supplementary Table S3 Weighting scale of items in the Coefficient of Sense (Cs)in the modified Delphi method**

|  |  | **Influence** |  |  |  |
| --- | --- | --- | --- | --- | --- |
| **Expertise field familiarity *** | **Strongly Familiar** | **Quite Familiar** | **Familiar** | **Less Familiar** | **Unfamiliar** |
| Child injury epidemiology | 0.5 | 0.4 | 0.3 | 0.2 | 0 |
| Disease monitoring methods | 0.2 | 0.15 | 0.1 | 0.05 | 0 |
| Clinical of child injury | 0.1 | 0.1 | 0.05 | 0.05 | 0 |
| Epidemiological research methods | 0.1 | 0.05 | 0.05 | 0.025 | 0 |
| Statistical analysis methods | 0.05 | 0.05 | 0.05 | 0.025 | 0 |
| Hospital information system | 0.025 | 0.025 | 0.025 | 0.025 | 0 |
| Construction of electric health records | 0.025 | 0.025 | 0.025 | 0.025 | 0 |
| Total | 1 | 0.8 | 0.6 | 0.4 | 0 |
